# The Genetic Basis Underpinning Sexually Selected Traits across Different Animal Lineages: Are There Genetic Mechanisms in Common?

**DOI:** 10.3390/ani14060841

**Published:** 2024-03-08

**Authors:** Ji Hyoun Kang

**Affiliations:** Korean Entomological Institute, Korea University, Seoul 02841, Republic of Korea; jihyounkang@korea.ac.kr

**Keywords:** co-option, evolutionary novelty, male ornament, male-specific trait, sexual selection

## Abstract

**Simple Summary:**

Sexual selection, through female choice or male–male competition, plays a crucial role in evolutionary diversification and speciation. While the evolutionary benefits and history of traits influenced by sexual selection are well-studied, the molecular genetic mechanisms of their development are less explored. Recent advances in genomic technologies such as RNA-Seq have shed light on the genetic basis of these traits across diverse taxa. This review compiles data on the genes and genetic processes involved in the development of sexually selected traits, revealing a common genetic architecture across different lineages. It highlights the frequent use of pre-existing genetic networks (i.e., gene network “co-option”) in the evolution of these traits, suggesting the repeated involvement of specific genes or gene sets in various sexually selected traits. Information on the genetic regulation of the development of sexually selected traits is valuable in providing a complete picture of their origin and evolution.

**Abstract:**

Sexual selection involving female choice or female preference (‘inter-sexual’ selection) and/or male–male competition (‘intra-sexual’ selection) is one of the key mechanisms for evolutionary diversification and speciation. In particular, sexual selection is recently suggested to be an important mode to drive the evolution of the “novel” phenotype (i.e., “evolutionary novelty”). Despite extensive studies performed on sexually selected traits or male-specific ornaments (or weapon-like structures) with respect to their evolutionary origin, history and fitness benefits, relatively little is known about the molecular genetic mechanisms underlying their developmental process. However, with advances in genomic technologies (including whole transcriptome analysis using Next Generation Sequencing [NGS] techniques; RNA-Seq), progress has been made to unveil the genetic background underpinning diverse sexually selected traits in different animal taxa. In the present review, empirical data on the genes, genetic mechanisms, or regulatory pathways underlying various sexually selected traits were compiled to explore whether “common” genetic architectures shape the development and evolution of these traits across evolutionarily distant animal lineages. It is shown that the recruitment of the pre-existing genetic network for a new purpose (i.e., gene network “co-option”) is rather widespread in the development and evolution of sexually selected traits, indicating that particular genes or gene sets are repeatedly involved in different sexually selected traits. Information on genes or genetic mechanisms regulating the development of sexually selected traits is an essential piece to complete a whole picture of the origin and evolution of sexually selected traits.

## 1. Introduction

Exaggerated male ornaments or weapon-like structures in various animal taxa, such as peacock’s tails and beetle horns, grasp peoples’ attention because of their extravagant appearance (coloration or morphology). Ever since Darwin described sexual selection, biologists have been fascinated by the origin and evolution of exaggerated male ornaments since they are thought to be subject to a “special” type of evolutionary process. [1]. These male-specific traits are believed to be sexually selected, meaning that they are beneficial for mating success by attracting mates or winning over rivals, although exaggerated traits can be costly and have possibly detrimental effects on fitness due to the action of natural selection (e.g., vulnerability to predators; see Zahavi, 1975 [2] and Endler, 1980 [3]. Female ornaments, given their reduced expression compared to those of males, can also be adaptive as selection can act on them [4,5,6,7]. Instead of rather a simple explanation of this trade-off between sexual and natural selection, the evolution of sexually selected traits is suggested to be much more complex, and has, thereby, become an interesting topic in evolutionary biology [8].

In general, two mechanisms are known to drive the evolution of sexually selected traits: inter-sexual selection (mate choice or preference) and intra-sexual selection (male–male or female–female competition) [9]. Exaggerated male secondary sexual characteristics are often the evolutionary outcomes of female preference to choose their mates [10]. Darwin hypothesized that female choice (or preference) leads to the evolution of male sexual ornaments [11], and many studies have provided empirical evidence supporting this idea (e.g., [12]) extreme male tail length in a widowbird [13]. These elaborated male ornaments might be used by females as a signal of male quality for choosing mates [14] in many animal taxa, including birds, flies, beetles, fish, ungulates, and crustaceans [15,16]. The sizes of male ornaments often matter for sexual selection in several species. For example, wing patch size is the target of female choice in collared flycatchers [17]. It was also shown in barn swallows that male ornament size acts as an “honest” signal by reflecting their offspring’s longevity [12]. Stalk-eyed flies showed a positive association of male reproductive morphology with enhanced fertility [18]. The length and darkness of a lion’s manes are signals for male qualities such as nutrition and fighting success [19]. These sexually selected traits, particularly color traits, can also play an important role in a social context (i.e., non-sexual social selection) if they confer social advantages (e.g., competition for non-reproductive resources and social status) to bearers [20,21]. When social signals (behavior) carry information about the signaler’s social status, such as their fighting ability, behavioral responsiveness becomes adaptive, impacting the receiver’s fitness as well [22]. In white-eyed bulbuls, mate choice tests revealed that females preferred unfamiliar males with increased yellow ornamentation, suggesting that both ornamentation (yellow ventral patch) and social familiarity could influence mate choice [21].

Some exaggerated male traits, such as claws in fiddler crabs [23], the horns of giant rhinoceros beetles [24], and antlers in red deer [25], are used as a weapon to deter rivals for mating competition. Exaggerated sexually selected traits are usually associated with individual variation in the traits based on age, size, nutritional condition, and genotype since their expressions are highly condition-dependent [15,16]. Mate choice based on these traits can provide genetic benefits to the choosing parents [26]. In the ‘good gene’ hypothesis, individuals possess alleles that can enhance the survival and reproductive success of their offspring through mate choice. Moreover, individuals can increase their fitness by choosing mates based on genetic compatibility (‘incompatibility avoidance’ and ‘genetic compatibility’) [27].

Female ornaments, with their expression often reduced, have long been perceived as non-functional. However, it is currently recognized that female ornaments can be adaptive, given that selection can act directly on female ornamentations [4,5,6,7]. For example, the male preference for the more sexual behavior for the coloration of female ornamental throat patches was experimentally confirmed in a blue throat [4]. It was also found that there were no sexual differences between males and females in the positive associations of ornament elaboration with quality and fitness across bird species [7]. Recently, male mate choice and the role of female ornaments have received attention for their significant contribution to male mate choice and inter- and intra-sexual selection dynamics [28,29,30]. Schlupp (2018) [28] particularly highlights the prevalence of male mate choice in live-bearing fishes (Poeciliidae), emphasizing its association with variations in female quality, such as fecundity. However, empirical data on female competition and ornamentation remain relatively scarce compared to male–male competition and male sexually selected traits. Although direct evidence is limited, two case studies suggest the potential for male mate choice based on female ornamentation. For instance, male Sceloporus lizards adjust their reproductive investment based on the size of female ornaments [29]. The relationship between aggression and female ornamentation has been explored in sticklebacks [30]. Moreover, research suggests that male mate choice, coupled with female–female competition and preference for female ornamentation, plays a crucial role in driving the evolution of ornamental traits [28]. Notably, female ornaments may impose less fitness costs compared to male ornaments, as reduced female fitness can also impact male reproductive success [31]. While female competition tends to be more indirect, the scarcity of direct observational data makes it challenging to assess its impact on sexual selection dynamics [32]. Despite these challenges, investigating female competition and ornamentation is essential for a comprehensive understanding of the role of mate choice in sexual selection.

The notion that male exaggerated ornaments are considered “evolutionary novelties” has brought interesting questions about their origin and evolutionary history, including lineage-specific loss and gain [24]. Evolutionary novelties or novel traits can be described as structures or characters that are not homologous to any structures that existed in ancestral lineages or any structures of the same species [33]. According to this definition, novel characters are considered genuine additions to an organism’s body plan, which cannot be traced back to an ancestral state. These traits are newly derived, and therefore, the evolution of a new character identity is required [34]. Novel functional capabilities (e.g., flight, vision) or novel structural elements (e.g., hair and horn in mammals, scales in reptiles) are two categories of evolutionary novelties when the developmental origin of novel body parts is considered in this regard [35]. On the other hand, novelties can also be considered “key innovations”, which are radically modified pre-existing characters, but their origin can be traced. Key innovations are sufficient to initiate the diversification of a lineage and are correlated with the evolutionary success of a newly appeared group of animals [36]. Therefore, it is important to view the evolution of male exaggerated traits as the de novo origination of traits, as well as the morphological changes in existing ones [34].

Despite extensive studies performed on sexually selected traits in terms of their evolutionary origins, history, and fitness benefits, little is known about the molecular mechanisms underlying the development of exaggerated male ornaments and sexually selected traits. An understanding of their developmental processes is essential to identify the origin of sexually selected traits (secondary sex-specific traits) [37]. In particular, unique developmental and evolutionary identities can be better understood by an investigation of the origin and divergence of novel gene regulatory networks contributing to morphological innovations [35]. If we know the genetic mechanisms of the origin and evolution of exaggerated male traits, then it will also help to understand the genetic mechanisms underlying evolutionary novelties. Information on genes or genetic mechanisms regulating the development of sexually selected traits (male exaggerated ornaments) is an essential piece to complete the whole picture of the origin and evolution of these traits.

While many studies have focused on the determination of genetic variation responsible for sexually selected traits (reviewed in [38]), research efforts on the identification of ‘causal’ or associated genes or their regulations underlying these traits are very limited. Recently, however, more and more studies about genes or genetic pathways underlying sexually selected traits have been performed in various distantly related animal lineages (e.g., sword in swordtails [39,40], horns in beetle species [41,42,43,44], antlers in deer [45,46,47,48], plumage coloration in birds [49,50], and eye span in stalk-eyed flies [51,52,53]). Advances in genomic technologies (including whole transcriptome analyses using Next Generation Sequencing [NGS] techniques) have facilitated research in this field at the genome-wide level. However, few attempts have been made to compare the genetic mechanisms or genetic backgrounds of sexually selected traits among different animal taxa. With this accumulating information, we are now able to compare genes and genetic pathways among different sexually selected traits across different taxa in order to test whether common (or similar or completely different) genetic mechanisms contribute to the development of these traits. If they share common or similar genetic mechanisms, then it provides the basis of knowledge that different sexually selected traits evolve through common molecular mechanisms. In the present review, I focus on the specific genes or genetic pathways involved in sexually selected traits, using recent emerging empirical data with NGS. The goals of this review are two-fold as follows: (1) to summarize empirical data on the genetic mechanisms underlying sexually selected traits and (2) to explore whether common (or shared) genetic mechanisms shape the development or evolution of sexually selected traits across evolutionarily distant animal lineages. Although it is difficult to identify the target (or causal) genes or genetic regulations underlying these traits by comparing expression genetic data, in doing so, I aim to suggest a framework or guide for future research to look deeper into the genetic basis underlying the origin and evolution of these traits. It would allow us to obtain a starting point to understand the complex of the conserved genetic mechanisms of evolutionary novelties.

## 2. The Genetic Basis of Various Sexually Selected Traits (SSTs) or Exaggerated Sexual Ornaments in Animal Taxa

Various sexually selected traits or sex-specific ornaments can frequently be observed in nature from diverse animal groups such as insects, fish, birds, and mammals. Examples include the horns in beetles [24], eye-span in stalk-eyed flies [54], sex combs in fruit flies [55], plumage coloration in birds [56], and antlers in deer [57]. Despite accumulating evidence of their crucial ecological role, little is known about the genes or genetic pathways underlying these traits, limiting progress in our understanding of the developmental origin of those evolutionary novelties. Recently, however, some progress has been made to identify a handful of (candidate) genes or genetic networks that might account for sexually selected traits. Here, several sexually selected traits that have been studied based on their genetic mechanisms or genetic pathways are listed (Table 1).

### 2.1. Sexually Selected Traits on Flies

The development and evolution of sexually selected male ornaments in insects have previously been reviewed [81]. The most intensive investigations on the gene expression or genetic mechanisms of sex-specific or sexually selected traits have been performed for fly species in insects. Stalk-eyed fly species have evolved male-exaggerated hypercephaly, known as “eyestalks”, which are the lateral projections of the head capsule [51]. Female diopsid flies use this trait as an indicator of male quality, and eyestalk size shows a large amount of interspecific variation [51]. With respect to its genetic regulatory architecture, the expression of *hedgehog* (*hh*), *wingless* (*wg*), *engrailed* (*en*), and a transcription factor, *defective proventriculus* (*dve*), as their *Droshophila* homologs were found in eye-antennal discs in stalk-eyed flies [51,58] (Table 1). Using EST (Expressed Sequence Tag) sequencing and microarray analysis, a study of eye-antennal imaginal discs in stalk-eyed flies revealed several candidate genes, such as *crooked legs* and *cdc2* [54]. Gene expression patterns in the developing tissues of the eyestalk indicate the potential role of gene duplication in the evolution of sex-specific traits [82].

Sex comb in males is another extensively studied, sexually dimorphic trait in *Drosophila* species, although it was suggested not to directly relate to sexual selection [83]. This trait is used for males to grasp the female abdomen and genitalia for their successful copulation. Several genes are found to be involved in the development of the sex comb. *Dachshund* (*dac*), which is known to have a conserved function in sensory organs and appendage development in insects, is shown to be involved in sex-comb development [61]. Candidate gene approaches identified *scr* and *dsx* [59], which also contribute to the sex-comb development in *Drosophila* [62]. Sex-determining genes such as *daschund* (*dac*) and homeobox genes have also been found to be expressed during their development [59,61,64].

### 2.2. Exaggerated Male Traits in Beetle Horns

Several seminal studies uncovered the novel genetic mechanisms underlying the rhinoceros beetle horns as a sexually selected male weapon. Beetle horn has become one of the famous examples of male exaggerated ornaments because of its magnificent size relative to their body and extraordinarily high levels of interspecific variation in terms of size and shape. It is a sexually selected trait through female choice and is also used as a weapon for male–male competition. Using combined analyses of comparative phylogenetic studies on horn evolution with developmental investigations on horn growth [24,67,84], the “evolutionarily labile horns” hypothesis was proposed. All modern phylogenies suggest that the gain and loss of horns are labile during evolutionary history, and their form sometimes changes rapidly and dramatically [84]. They found that genetic changes on the domain, such as *hh*, *wg* and *dpp* signals, determine the precise locations of the horns’ outgrowth. Even subtle changes to the genes involved in the limb-patterning pathways can lead to drastic changes in horn forms and shapes [84]. The same research team published seminal studies on the genetic mechanisms of the beetle horn and revealed novel functions of the genes involved in the sexually selected trait in beetles. Insulin signaling pathways, a major regulator for tissue growth and body size [85,86], have been suggested as a candidate genetic pathway for the evolution of the beetle ‘horn’ [84]. They further showed a significantly higher sensitivity of cells to insulin/the insulin-like growth factor (IGF) in beetle horns (weapons) compared to other traits (genitalia and wings) in the rhinoceros beetle [69]. This increased cellular sensitivity to insulin/IGF pathways is suggested to cause extreme growth because it acts as a reliable signal of better male quality, or it is simply a by-product of the growth mechanisms [69]. Furthermore, *insulin receptors* (*InRs*) are found to be responsible for polymorphic horn developments in sexually dimorphic male-horned beetles [70,87]. The role of the insulin signaling substrate *chico* and the ecdysone response element *broad* head for horn length was shown in the knockdown experiment [87]. A conditional expression of a nutritional state or physiological condition in exaggerated (head and thorax horns) traits was observed using RNA-seq in the Asian rhinoceros beetle [44].

### 2.3. Swords in Swordtail Fish

“Sword” in swordtail fish in the genus *Xiphophorus* is one of the well-known examples of sexually selected traits in fish. Some *Xiphophorus* species, such as swordtails, but not others, like platies, have a male-specific trait, the “sword”, that is an elongated colored extension of the ventral rays of the caudal fin. Some species have very long, extended, colorful swords that can be even longer than the body of the males [88,89]. The sword is an evolutionary novelty in this genus, and its origin and evolutionary history have extensively been investigated in a phylogenetic context [90,91,92,93]. The origin of the sword has been under debate for several decades. One of the hypotheses explaining the origin of the sword is the pre-existing bias hypothesis that a female preference (or sensory bias) for swords already existed before the appearance of swords, which drove the evolution of the sword in several swordtail species [94,95]. This hypothesis is supported by the fact that several platy fish females, of which males do not carry a sword, still show a preference for males with artificial swords [94]. Comprehensive phylogenetic analyses of *Xiphophorus* suggested that the sword existed in a common ancestor in this genus and was lost secondarily in platies multiple times independently [91,92,93].

Genes or genetic pathways involved in the development of the sword have been identified in several studies. The candidate gene approach revealed that several genes, such as *msx* and *fgfr1*, are expressed in the developing sword under hormone treatment in a swordtail species, *Xiphophorus hellerii* [74,75] (Table 1). Kang et al. [96] investigated gene expression changes in the developing sword at the whole transcriptome level using high-throughput RNA-Seq in the swordtail, *X. hellerii*. This study provided a catalog of candidate genes to understand the architecture of gene regulatory networks of the development of the sword. A large number of differentially expressed genes (1784) in hormone-induced swords highlight the massive changes that are taking place during the development of the sword [96]. Interestingly, many embryonic developmental genes were involved in sword development, and approximately 70% of these differentially expressed genes were shared by another male-specific and evolutionary older trait, gonopodium. These findings suggest that genetic networks are “co-opted” during the development and evolution of gonopodium and are also subsequently deployed in the later evolution of another novelty, the sword [96]. Recently, a transcriptome analysis of the sword of *X. hellerii* also revealed a series of genes responsible for pigmentation (*xdh*, *tyr*, *myrip*, *asip*), vascularization (*agtr1*, *angptl5*), and fin-ray rigidity-related genes [40].

Furthermore, genes are often expressed in neurons, and Ca^2+^ signaling is differentially expressed in the sword regions compared to control fin regions [40]. Several transcriptional factors, such as *homeobox protein six2a*, *hoxb13a*, *tbx3a*, and *pax9*, were also suggested as candidate genes to regulate sword formation in a quantitative manner. More interestingly, a channel protein gene, *kcnh8*, abundantly expressed in the brain, was suggested as a sword-developing gene by transcriptome analysis combined with QTL mapping [40]. A companion study also identified the up-regulated expression of *kcnh8* on regenerating caudal tissues in other *Xiphophrous* species (*X. birchmanni*, *X. malinche*) [39]. These genetic mapping approaches, combined with transcriptome analysis, highlight a polygenic basis for the diversity of the sexually selected sword trait.

### 2.4. Colorful Sexual Traits

Coloration on sexual ornaments is often regarded as the evolutionary outcome of sexual selection since it represents an honest signal of the individual quality of the mate (e.g., male condition and genetic quality) and can, thus, be used by females for choosing their partner [21,97]. Examples include plumage and melanin colorations in birds [98,99,100,101] and pigmentation patterns in African cichlid fishes [102,103] and guppies [76]. In particular, a carotenoid pigment that is responsible for yellow, orange, and red colorations has been suggested as an indicator of various health conditions reflecting male quality in many fish and birds [104,105].

The genetic mechanisms of the different pigmentation-based body colorations have been investigated. Recent transcriptome approaches identified several potential genes, such as the *coatomer protein complex* and *subunit zeta-1* (*copz-1*), that might be involved in coloration [106] and melanophore maintenance in cichlid fish [107]. It has been shown that black ornaments of guppy males develop under the effect of *colony-stimulation factor 1 receptor* a (*csf1ra*), which mediates the xanthopore–melanophore interaction [108]. Transcriptomic studies of carotenoid pigmentation provided a candidate gene list, including the expression of *Eorix* proteins in carotenoid-signaling bird species [109]. Genes responsible for beak color in zebra finches were identified using QTL (Quantitative Trait Loci) analyses [110], and also genes for melanin-containing organelles (melanosomes) were found [111]. Hox gene pathways were found to be involved in sexually selected pigmentations in *Drosophila* species [112,113]. In wild guppy populations, multilocus heterozygosity (MLH) was suggested to be a significant predictor of the orange spot in males as a sexually selected trait by female preference [114].

## 3. Common Genetic Architecture among Sexually Selected Traits

Recent investigations on diverse sexually selected traits or exaggerated male ornaments provide an interesting insight into their genetic backgrounds or genetic mechanisms. Intriguingly, accumulating information about genes or genetic pathways and accounting for sexually selected traits/exaggerated ornaments revealed several groups of genes that are commonly involved across distantly related animal lineages. For example, conserved genes are expressed in eye-antennal discs in both *Drosohophila* species and stalk-eyed flies [51]. Water striders (*Aquarius remigis*) and *Drosophila* species diverged around 371.9 mya ago (www.timetree.org, accessed on 7 January 2024) [115], but they still exploit the same genetic mechanisms to develop sexually selected traits. Although the shared genes might not be regulated in the same way across traits and taxa, the findings of the common genetic architecture underlying diverse sexually selected traits in different animal lineages provide valuable insights for a better understanding of their developmental origin and evolutionary history.

### 3.1. Signs of Co-Option

The recruitment of pre-existing genetic network systems for new purposes using the “gene network co-option” has been suggested to be a usual method for the development and evolution of morphological novel traits [116,117,118]. Several empirical studies showed that the co-option of certain gene networks (i.e., developmental gene networks) underlies the developmental origin of novel traits. For example, the co-option of genes or regulatory networks related to anteroposterior head patterning for proximodistal appendage patterning in fruit flies [119], eye-developmental genes (i.e., *optix*) for red patches of pigmentation on butterfly wings [120] and also for various wing scales as a novel trait contributing to speciation [121] have been identified. Similar patterns were also observed in the development of male-exaggerated ornaments in other animal groups [68]. Moczek and Rose [68] showed that limb patterning genes (distal-less and homothorax) that play an essential role in the limb development of other insect species regulate horn development in beetle horns, although horns are not modified at all from mouthparts or limbs. Many studies on the genetic basis of sexually selected traits found that a handful of specific genes are repeatedly involved in different sexually selected traits. The co-option of the *Hox* complex has been shown to contribute to cephalopod-specific organs [122]. *Hox* genes are well-known principal transcriptional regulators of animal body regionalization during embryonic development [55,60]. *Hox* genes have also been suggested to be key players in the development and evolution of novel complex traits such as male genitalia (i.e., imaginal discs) [123] and secondary sexual traits—sex combs in *Drosophila* species [64] and beetle horns [65]. The transcriptomic profiling of the sword in *Xiphophorus hellerii* showed that many *hox* genes (i.e., *dlx*, *lhx9*, *satb2*, *zhx2, etc.*) are involved in its development [96]. Another *hox* gene, distal-less (*dlx* or *dll*), which is known to be related to morphological development, was shown to regulate the development of beetle horns [68], antenna in water striders [124], the mandible in stag beetles (*cmdsx*) [125], eyespot size in butterflies [126] and swords in swordtails [96]. This gene is differently expressed according to the species, sex, body regions, and size in beetle horns [68]. *Dlx* paralogs have also been found as candidate genes for evolutionary innovations in cichlid fish [127]. An analysis of male sex comb regions showed that *dlx* expression is significantly correlated with other *hox* genes [60]. *Scr* also belongs to another *hox* gene family, and its role is known to regulate the segment identity in many insect groups, including *Drosophila melanogaster* [128,129]. *Scr* is also found to be expressed in the development of various sexually selected traits [64,65,66,74,130].

Bone morphogenetic proteins (*bmp*) are known to play an essential role in many different but important developmental pathways [131]. The expression of *bmp-3b* [77] and *bmp2* [78] is detected in deer antlers, which is a male weapon used in male–male competition for mating. The pleiotropic effects of *bmp2* and *hao1* have been identified in the comb mass, a sexual ornament in chickens, via QTL mapping [72]. A comprehensive transcritpomic analysis using RNA-Seq found the expression of *bmp1* in the developing sword of swordtail fish [96]. Moreover, several *bmps* are involved in the development of evolutionarily and ecologically important traits, such as the beak of Darwin’s finch, which is a classic example of adaptive radiation (i.e., *the* correlation of expression of *bmp-4* for beak morphology) [132]. These empirical data support the co-option of *bmps* in the expression or development of sexually selected or exaggerated traits. The genes found in the development of diverse sexually selected traits or exaggerated male ornaments are indeed key regulators for embryonic development and also for the development of non-sexually selected (normal) body parts. Therefore, I hypothesize that co-option plays an important role in the development and evolution of sexually selected traits in general.

### 3.2. Sex Determination and Sex-Biased Genes

It has been suggested that the coordination of sex-specific development assists in the evolution of sexual traits and the gene regulatory network governing sexual development [38]. Sex-determination systems are enormously diverse across different taxa, ranging from single-factor genotypic sex (e.g., XX/XY) to environmental sex determination (e.g., temperature or pH controlled) [133], but their downstream components are known to be generally more evolutionarily conserved [37]. A few reported genes related to a function of sex determination are known, including a *sex-determining region on Y* (*sry*) in mammals, *dmrt1* (doublesex and related transcription factor 1) on the Z chromosome in birds, *dmy*, *gsdf*, and *amhr2* in fish [134,135,136,137,138,139].

It is also important to note that variations in the localization of these specific DNA sequences (e.g., LINE1) across different chromosome types play a significant role in sex-determination processes and the expression of sex-biased genes. These sequences may be exclusively localized on sex chromosomes (e.g., Line 1 in the X-chromosome) [140,141,142], solely on autosomes [141], or present on both autosomes and sex chromosomes [142]. For instance, Bailey et al. (2000) [140] observed a twofold enrichment of long interspersed repeat element (LINE)-1 (L1) on the X chromosome, which influences X-inactivation.

A majority of sex-determination genes are known to be typically involved in the development of primary sexual traits (i.e., gonad). However, more and more evidence is accumulating that those genes are also activated in the development of “secondary” sexual traits (i.e., sexually selected traits). For example, *doublesex* (*dsx*), which is a well-known key regulator for the sex-determining cascade gene in insects, is found to regulate the development of sexually dimorphic traits in fruit flies, mammals, birds, and fish [37,63]. *Dsx* functions as a regulator, controlling various aspects such as dimorphisms between sexes, morphs, and species in the development of beetle horns [41,71]. This gene is also involved in the development [128,143] and the sex-specific expression of the sex comb in *Drosophila* species. Expression patterns of *cyclommatus metallifer dsx* (*cmdsx*) in the sex-specific mandible growth in male stag beetle were shown to be mediated by juvenile hormone (JH) signaling pathways [125].

Several *sex-determining regions on Y* (*sry*) are generally known to be involved in the development of primary sexual characters (i.e., testis development). Nevertheless, those genes are also found to be expressed during the development of secondary sexual characters. Several *sox* genes (*sox* 2, 3, 5, 9, and 10) were shown to be involved in sword development [96]. The transcriptome analysis of Chinese sika deer antlers identified the expression of *SRY-box 9* (*Sox9*) during rapid growth in antler development [45]. In particular, *Sox9* (*sry-box 9*) is a well-known sex-determination gene [144]. Other genes, such as *Inhbb* [145], *cyps*, and g*ps7*, *8* [146], which previously demonstrated sex-specific divergent expression patterns in other animal groups, were also differentially expressed in the developing sword in swordtail fish [96].

### 3.3. Insulin Signaling Pathway

The higher sensitivity of cells to insulin/insulin-like growth factor (IGF) signaling pathways in a male-exaggerated trait (e.g., horn) compared to other body parts (e.g., genitalia and wings) in rhinoceros beetle horns suggests that IGF signaling genetically controls horn development and generates an honest signal of male nutritional conditions [69,147]. It has been suggested that the extreme growth of horns in rhinoceros beetles is a by-product of growth mechanisms and demonstrates an important role in IGF pathways in horn development. Several growth factor-related genes are found to be expressed in the development of diverse sexually selected traits. Growth factor-related genes such as *transforming growth factor*, *beta 3* (*tfgb3*), *insulin-like growth factor 2* (*igf2*), *insulin-like growth factor binding protein 4* (*igfbp4*), and *IGF-like family receptor 1* (*igflr1*) were differently expressed in the developing sword in swordtail fish (*Xiphophorus hellerii*) compared to other tissues [96]. GO terms such as growth factor activity and the response to growth factor stimulus were also enriched in the developing sword. A comprehensive transcriptome study in Sika deer antler found that *igf* II is significantly highly expressed [47]. Transcriptome analysis of the guppy (*Poecilia reticulata*) showed that male-biased-expressed genes in tails are enriched with the GO term related to insulin receptor binding [148]. The expression of genes involving the insulin and IGF signaling pathways in various male-exaggerated ornaments across several animal groups might indicate that different sexual traits also use the same genetic mechanisms for their development processes, as suggested in beetle horns.

### 3.4. Steroid Hormone-Related Genes

The role of steroid hormones (e.g., androgens, estrogens, and glucocorticoids) in the expression or development of sexually selected traits has been extensively examined in several taxa. A positive relationship was found between the levels of steroid hormones and the sizes of sexual ornaments. For instance, badge size in house sparrows is associated with levels of testosterone [149], and the size of a melanin-based black bib in male house sparrows is associated with glucocorticoid receptors [150]. The regulation of testosterone in the development of deer antlers was also identified [151]. Whether levels of testosterone and corticosterone predict the dewlap size in males of green anole lizards (*Anolis carolinensis*), which is a predictor of bite-force capacity, was also tested [152]. The relationships between the hormone levels and the ornament size in this lizard species vary according to their body size (e.g., heavy-weight and light-weight) [152].

It has been shown that sex hormone-related genes are involved in the development of various sexual ornaments both directly and indirectly. Androgen-modulated expression has frequently been identified in several sexually selected colorations such as red gular pouch coloration (skin ornament) in a frigate bird [153], sexually dimorphic facial hair coloration in red-fronted lemurs [154], an enlarged red eye ring (more pronounced in males) in diamond doves [155] and male plumage color in red-backed fairywrens [156,157]. However, direct examples showing the expression of genes involved in androgen pathways in sexually selected traits or male ornaments are rather sparse. The expression of *androgen receptor β* (*arβ*) was found in the developing swords in *Xiphophorus hellerii*, which is a swordtail species possessing a long, colorful sword [74,75,96].

Estrogen levels have an influence on the regulation of sexual ornaments in males as well as female-specific ornaments. The sex-specific regulation of estrogen pathways rather than androgen or insulin growth factor signaling pathways is suggested to be a primary regulator factor in affecting male-biased polymorphism with long faces in male Anolis lizards [158]. Similarly, several estrogen-related genes were found to be up-regulated in the male-specific traits of the sword in swordtail fish [96]. Previously recognized genetic mechanisms underlying sexual dimorphism need to be further examined [155]. Steroid hormone signaling pathways are one of the intriguing future avenues for genetic mechanisms underlying male-specific sexual traits.

### 3.5. Other Reported Common Genes

Besides the genes or genetic pathways mentioned above, several other genes are commonly expressed in sexually selected traits among distantly related lineages. For example, *crooked legs* and *cdc2* are expressed in the eye-antennal imaginal discs of stalk-eyed flies [54]. Zinc finger proteins, the homologs of *crooked legs*, and several *CDCs* (e.g., *CDC7*, *16*, *20*, *27*, and *34*) are also up-regulated in the developing swords of *X. hellerii* [96]. *Forkhead* (*fkh*) genes (e.g., *FOXs*), which are the natural targets of *sex combs reduced* (*Scr*), are expressed in the sex combs of *Drosophila* species [159] and also upregulated in the sword of the swordtail [96].

## 4. Transcriptome Profiling of Sexually Selected Traits or Male Ornaments

Deep sequencing techniques offer an opportunity to explore the genetic mechanisms or regulatory architecture underlying evolutionarily or ecologically interesting traits such as adaptive phenotypes, including sexually dimorphic characters or sex-specific (often male-specific) ornaments in non-model organisms. Transcriptome studies on sex per se have increasingly been published, and they provide a fundamental resource for sex-specific or sex-biased gene expression patterns in various animal taxa [148,160,161,162,163]. This information on sex-specific gene expression data can also be used as a valuable resource to explore the genetic regulatory background underpinning the development of sexually selected traits or male-specific ornaments. Although only a few transcriptome studies that look directly into these sexually selected traits are currently available (relative to transcriptome studies on sex per se), it is worthwhile to review them to obtain an overview of transcriptome profiles of sexually selected traits (Table 2). One general pattern is that most transcriptomes expressed in sexually selected traits show a great number of genes having undergone changes in expression during their development. For example, for transcriptome analysis on the development of sika deer antlers, 5573 genes (out of 16,905 significantly changed transcripts) were differentially expressed at two different developmental stages [47]. A developing sword in swordtail fish showed that 1782 genes are differentially expressed compared to the control fin rays under hormone treatment conditions, and several sword-specific genes were also identified [96].

Transcriptome profiles can also provide information on trait-specific gene expression patterns or particular genetic pathways that are mostly represented in traits of interest. For instance, the transcriptome analysis of the sika deer antler revealed that genes and genetic pathways related to protein synthesis and translation (i.e., elongation factors) are most significantly changed during development [47]. Sword transcriptome showed that embryonic organ development, sexual character development, and coloration genes were significantly highly expressed [96]. Kang et al. (2015) further suggested co-opted genetic networks for the development of swords and another male-specific sexual trait, gonopodium [96]. A transcriptome analysis combined with QTL mapping suggested that a brain gene, channel protein gene *kcnh8*, was recruited for the evolution of the sexually selected male ornament of the sword [40].

Some genes or genetic pathways shared between transcriptome profiles of two different sexually selected traits in distantly related lineages have also been identified. In the transcriptomes of two sexually selected traits, the sword in swordtails [96] and the tail in guppies [148], several GO terms such as plasma membrane (GO:0005886), the cellular biogenic amine metabolic process (GO:0006576), carbohydrate transport (GO:0008643), neuropeptide signaling pathway (GO:0007218) and melanosome (GO:0042470) are commonly found. Interestingly, it was also found that many genes or groups of genes aforementioned on other sexually selected traits such as the color gene [*xanthine dehydrogenase* (*xdh*) and *premelanosome protein (pmel*)], hox genes [*ALX homeobox 4* (*alx4*)], hormone gene [*adrenoceptor alpha 2B*], growth factor-related genes [*growth hormone receptor* (*ghr*), *insulin receptor substrate 2* (*irs2*), *insulin-like growth factor 2 mRNA binding protein 3* (*ifg2bp3*)], sex-determining genes (*sox8*, *9*, *10*) and *pax9* are all commonly found in both sexually selected traits. Transcriptome investigations on the sexually selected traits have still been limited. However, emerging transcriptome analyses could provide important resources for future research on investigating conserved genes or genetic pathways underlying sexual traits in distantly related species at the whole transcriptional level. This information is very useful to select candidate genes or genetic pathways to investigate the genetic mechanisms in more detail and understand how sexually selected traits or male ornaments have arisen. Furthermore, accumulating comprehensive transcriptome data on these sexual traits could shed valuable insight into future research to understand the evolutionary origin and the genetic mechanisms of the development of evolutionary novelties more widely.

## 5. Conclusions

I reviewed the genes, genetic mechanisms, and regulatory pathways found in studies that investigated the genetic basis of sexually selected traits or exaggerated male (and also female) traits in diverse animal groups. While genetic (genomic) data on these traits are increasing with advanced sequencing technologies, the amount of information is not sufficient thus far to comprehend the whole story of the development and evolution of sexually selected traits. However, common genes and genetic architectures shared among different sexually selected traits across distantly related animal lineages provide a framework for future research on uncovering their origin and evolution. Further, commonly expressed genes are critical for future studies to identify upstream or downstream regulators of sexually selected or male exaggerated traits and also determine the transcriptional changes in these genes for different traits.

## Figures and Tables

**Table 1:** Genes identified as involved in sexually selected traits or male ornaments from studies using candidate gene approaches.

Trait	Organism	Genes	Features	References
Eye span (eye-antennal disc)	Salk-eyed flies (*Cyrtodiopsis dalmanni*, *C. whitei*, and *Sphyracephala beccarn*)	*Distaless* (*dll*), *hedgehog* (*hh*), *wingless* (*wg*), *engrailed* (*en*), and *defective proventriculus* (*dve*)	-	[51,53,58]
	Stalk-eyed flies (*Teleopsis dalmanni*)	*Crooked legs* and *cdc2*	EST (Expressed Sequence Tag) sequencing and microarray analysis	[54]
Sex comb	Fruit fly (*D. melanogaster*)	*Sex comb reduced* (*Scr*), *doublesex* (*dsx*)	Sex comb morphogenesis	[59,60]
		*Daschund* (*dac*) and *distaless* (*dll*)	Sex comb development	[61]
	Fruit fly (*D. mauritiana* and *D. simulans*)	*Dsx*	Sex comb divergence	[62,63]
	Fruit fly (*Drosphila* species)	*Scr*	-	[37,62,64,65]
Male-specific abdominal pigmentation	Fruit fly (*D. melanogaster*)	*Bric-à-brac* (*Bab*) and *dsx*	Genes from pre-existing dimorphic traits	[66]
Horn (weapon)	Rhinoceros beetle	*Hh*, *wg* and *dpp*	Determination of precise location of horn outgrowth	Reviewed in [67]
	*Onthophagus taurus* and *O. binodis*	*Dlx* or *dll*	Tissue-specific expression	[68]
	Rhinoceros beetles (*Trypoxylus dichotomus*)	*Insulin/insulin-like growth factor*, *chico*, *Broad*	Tissue-specific expression, RNAi-mediated knockdown	[69,70]
	Rhinoceros beetle (*Onthophagus taurus* and *O. sagittarius*)	*Dsx*	Morph-, sex- and species-specific development	[71]
Comb mass (sexual ornament)	Chicken (White Leghorn chickens and a Red Junglefowl)	*Bmp2*, *hao1*, *CHADL*	Pleiotropic effects	[72,73]
Sword	Swordtails fish (*X. hellerii*)	*Msx* and *fgfr1*	Hormone-induced features	[74,75]
Male black ornament	Guppy (*Poecilia reticulata*)	*Colony-stimulation factor 1 receptor* a (*csf1ra*) and *Kita*	Pigment pattern formation	[76]
Antler	Deer species	*BMP-3b*, *BMP2*, *ANXA2*, *APOD* and *TPM1*	-	[77,78,79,80]

**Table 2:** Recent genome-wide transcriptomic and QTL studies on sexually selected traits.

Trait	Organism	Features	Method	References
Horn	*Onthophagus* beetles	Development of horns	EST and microarray	[41]
	Horned beetle (*Onthophagus taurus*)	Whole body including horns	EST (454 pyrosequencing)	[42]
		Development of horns (head horns and thoracic horns)	Microarray	[164]
	Asian rhinoceros beetle (*Tryposylus dichotomus*) and dung beetle (*Onthophagus nigriventris*)	Phenotypically plastic traits (horn-biased gene expression)	RNA-Seq	[43]
		Exaggerated (head and thorax horns)	RNA-Seq	[44]
Legs	Water strider (*Microvelia longipes*)	Exaggerated legs in males (weapons)	RNA-Seq	[165]
Antler	Sika deer (*Cervus Nippon hortulorum*)	Endochondral ossification (ossification stages)	RNA-Seq	[48]
		Regeneration and rapid growth (antler’s tip)	RNA-Seq	[145]
		Differential developmental stages (60 and 90 days)	RNA-Seq	[47]
		Development of antler’s tip	RNA-Seq	[166]
Plumage coloration	Ruff (*Philomachus pugnax*)	Plumage coloration and mating strategies	RNA-Seq	[49]
	Bearded reedling (*Panurus biarmicus*)	Plumage coloration	RNA-Seq	[167]
	Red-backed fairywrens (*Malurus melanocephalus*)	Plumage coloration	RNA-Seq	[156]
Sword	Swordtail fish (*Xiphophorus hellerii*)	Development of male ornament and sexual organ under the hormone-treated conditions	RNA-Seq	[96]
	Swordtail fish (*Xiphophorus* species)	Sword	RNA-Seq, QTL	[39,40]

## Data Availability

The data presented in this study are available in article.
